# A cross‐lagged twin study of emotional symptoms, social isolation and peer victimisation from early adolescence to emerging adulthood

**DOI:** 10.1111/jcpp.13847

**Published:** 2023-06-06

**Authors:** Geneviève Morneau‐Vaillancourt, Olakunle Oginni, Elham Assary, Georgina Krebs, Ellen J. Thompson, Elisavet Palaiologou, Celestine Lockhart, Louise Arseneault, Thalia C. Eley

**Affiliations:** ^1^ Social, Genetic and Developmental Psychiatry Centre, Institute of Psychiatry, Psychology and Neuroscience King's College London London UK; ^2^ Department of Mental Health Obafemi Awolowo University Ile‐Ife Nigeria; ^3^ Research Department of Clinical, Educational and Health Psychology University College London London UK; ^4^ Department of Twin Research and Genetic Epidemiology, School of Life Course and Population Sciences King's College London London UK; ^5^ National Institute for Health Research (NIHR) Biomedical Research Centre South London and Maudsley Hospital London UK

**Keywords:** Anxiety, depression, peer relationships, twins, longitudinal studies

## Abstract

**Background:**

Emotional symptoms, such as anxiety and depressive symptoms, are common during adolescence, often persist over time, and can precede the emergence of severe anxiety and depressive disorders. Studies suggest that a vicious cycle of reciprocal influences between emotional symptoms and interpersonal difficulties may explain why some adolescents suffer from persisting emotional symptoms. However, the role of different types of interpersonal difficulties, such as social isolation and peer victimisation, in these reciprocal associations is still unclear. In addition, the lack of longitudinal twin studies conducted on emotional symptoms during adolescence means that the genetic and environmental contributions to these relationships during adolescence remain unknown.

**Methods:**

Participants (*N* = 15,869) from the Twins Early Development Study completed self‐reports of emotional symptoms, social isolation and peer victimisation at 12, 16 and 21 years old. A phenotypic cross‐lagged model examined reciprocal associations between variables over time, and a genetic extension of this model examined the aetiology of the relationships between variables at each timepoint.

**Results:**

First, emotional symptoms were reciprocally and independently associated with both social isolation and peer victimisation over time, indicating that different forms of interpersonal difficulties uniquely contributed to emotional symptoms during adolescence and vice versa. Second, early peer victimisation predicted later emotional symptoms via social isolation in mid‐adolescence, indicating that social isolation may constitute an intermediate pathway through which peer victimisation predicts longer‐term emotional symptoms. Finally, individual differences in emotional symptoms were mostly accounted for by non‐shared environmental factors at each timepoint, and both gene–environment and individual‐specific environmental mechanisms were involved in the relationships between emotional symptoms and interpersonal difficulties.

**Conclusions:**

Our study highlights the necessity to intervene early in adolescence to prevent the escalation of emotional symptoms over time and to consider social isolation and peer victimisation as important risk factors for the long‐term persistence of emotional symptoms.

## Introduction

Emotional symptoms, also referred to as internalising problems, comprise a broad range of anxiety and depressive symptoms, such as worry, fear and sadness (Caspi, Houts, Fisher, Danese, & Moffitt, 2023). Adolescence is a critical period for the development of emotional symptoms, with approximately 10% to 25% of adolescents suffering from severe clinically relevant anxiety and depressive symptoms (Lu, 2019; Racine et al., 2021; Tiirikainen, Haravuori, Ranta, Kaltiala‐Heino, & Marttunen, 2019). Emotional symptoms are associated with detrimental long‐term outcomes, including academic disruption and serious mental and physical health problems (Cohen, Edmondson, & Kronish, 2015; Johnson, Dupuis, Piche, Clayborne, & Colman, 2018; López‐López et al., 2020). Unfortunately, emotional symptoms often remain untreated and show continuity into adulthood, where they can escalate into full‐syndrome and recurrent anxiety and depressive disorders (Johnson et al., 2018; Kwong et al., 2019; Naicker, Galambos, Zeng, Senthilselvan, & Colman, 2013; Wolitzky‐Taylor et al., 2014). It is therefore crucial to identify the mechanisms by which emotional symptoms develop and persist during adolescence, as this will help identify young individuals at risk and inform early intervention strategies.

One pathway contributing to the continuity of emotional symptoms may be negative interpersonal experiences, such as peer victimisation. Peer victimisation affects 10%–35% of adolescents and is both a precursor and an outcome of emotional symptoms (Cook, Williams, Guerra, Kim, & Sadek, 2010; Modecki, Minchin, Harbaugh, Guerra, & Runions, 2014; Moore et al., 2017; Reijntjes, Kamphuis, Prinzie, & Telch, 2010). Several studies have examined the bidirectional associations between emotional problems and peer victimisation by using cross‐lagged models. Although many support a transactional model where emotional symptoms and peer victimisation mutually reinforce each other over time (e.g., Forrest, Gibson, Halligan, & St Clair, 2020; Smith, Hébert, Brendgen, & Blais, 2022; Zhu et al., 2022), others align with a symptom‐driven model, where emotional symptoms precede and perhaps evoke peer victimisation (e.g., Barzeva, Richards, Meeus, & Oldehinkel, 2020; Lee & Vaillancourt, 2019; Pabian & Vandebosch, 2016). There is also evidence supporting an interpersonal risk model, where peer victimisation predicts later emotional symptoms (Williford et al., 2012). These mixed findings could be explained by the fact that most studies relied on narrow definitions of anxiety and depressive symptoms and evaluated them separately. Yet, adopting a broader definition of emotional symptoms, especially within a longitudinal context, may more accurately depict the experience of adolescents affected by these problems. Anxiety and depressive symptoms often co‐occur, are characterised by sequential comorbidity (i.e., where one predicts the emergence of the other), share genetic risks and can be classified under a broader dimension of internalising problems (Caspi et al., 2023; Jacobson & Newman, 2017; Melton, Croarkin, Strawn, & McClintock, 2016; Middeldorp, Cath, van Dyck, & Boomsma, 2005). The few cross‐lagged studies that have assessed emotional symptoms broadly (i.e., both anxiety and depressive symptoms) consistently show that emotional symptoms are reciprocally associated with peer victimisation, thereby supporting the transactional model (e.g., Boyes, Bowes, Cluver, Ward, & Badcock, 2014; Smith et al., 2022).

In addition, other types of interpersonal experiences during adolescence, such as social isolation, may be involved in these reciprocal associations. Emotionally vulnerable adolescents often withdraw socially because of low mood, loss of interest, or worrying about social situations (Barzeva et al., 2020; Barzeva, Meeus, & Oldehinkel, 2019; Rubin, Coplan, & Bowker, 2009). They are also more likely to be rejected or excluded by their peers, who can be reluctant to initiate interactions with adolescents who exhibit negative emotions (Barzeva et al., 2020; Bowker, Nelson, Markovic, & Luster, 2013; Oh et al., 2008). Consequently, socially isolated adolescents are often viewed as vulnerable targets for peer victimisation (Acquah, Topalli, Wilson, Junttila, & Niemi, 2016; Sentse, Prinzie, & Salmivalli, 2017). In turn, their desire not to be exposed to peer victimisation might lead them to avoid social situations (Hutzell & Payne, 2012), or peers who are unwilling to affiliate with victims might reject them (Salmivalli, Lagerspetz, Bjorkqvist, Osterman, & Kaukiainen, 1996). This cascade of negative social experiences could ultimately aggravate emotional symptoms, as lacking positive peer relationships and opportunities to practise socialising often contributes to emotional symptoms (Barzeva et al., 2020; Matthews et al., 2016). As such, social isolation may be an intermediate pathway through which emotional symptoms lead to peer victimisation and vice versa.

Longitudinal twin studies have shed light on the mechanisms underlying these pathways by showing that genetic and environmental factors independently contribute to the associations between emotional symptoms, social isolation and peer victimisation (e.g., Matthews et al., 2016; Schaefer et al., 2018). Social isolation and peer victimisation do not occur randomly and are both heritable (30% to 70%), suggesting that genetic factors contribute to individual differences in these social difficulties (Fisher et al., 2015; Johansson et al., 2022; Matthews et al., 2016; Veldkamp et al., 2019). Evidence suggests a genetic overlap between emotional symptoms and negative peer experiences, meaning that a genetic disposition for emotional symptoms enhances the risk of being exposed to negative social experiences via gene–environment correlations (Brendgen, Zheng, Vitaro, Dionne, & Boivin, 2022). Furthermore, twin studies show that negative peer interactions increase emotional symptoms via non‐shared environmental pathways, providing evidence that these associations are unconfounded by pre‐existing genetic dispositions or by features of the familial environment shared by both twins (Arseneault et al., 2008; Schaefer et al., 2018; Silberg et al., 2016). Therefore, the mechanisms by which emotional symptoms are related to interpersonal difficulties involve a mix of gene–environment correlations and individual‐specific environmental influences.

Despite these findings, questions remain unanswered. First, most cross‐lagged studies on emotional symptoms solely focused on peer victimisation without considering other types of interpersonal difficulties and were conducted over short periods (e.g., 1 or 2 years). As such, it remains unclear whether social isolation acts as an intermediate pathway in the reciprocal associations between emotional symptoms and peer victimisation, and whether these associations persist over an extended period and across life transitions, such as from adolescence and into adulthood. Second, the extent to which genetic and environmental influences contribute to individual differences in emotional symptoms, social isolation and peer victimisation from adolescence to emerging adulthood, as well as to their relationships over this period, remain unknown. Documenting these genetic and environmental influences within a longitudinal perspective could enhance our understanding of the mechanisms underlying the persistence of emotional symptoms and its relationships with interpersonal difficulties over time.

The present study aimed to address these gaps by testing (a) the reciprocal phenotypic associations between emotional symptoms, social isolation and peer victimisation from 12 to 21 years old, and (b) the genetic and environmental influences and overlap of emotional symptoms, social isolation and peer victimisation at 12, 16 and 21 years old.

## Methods

### Participants

Participants were from the Twins Early Development Study (TEDS), a longitudinal cohort of twins in England and Wales (Lockhart et al., 2023). TEDS recruited more than 13,000 families from a national register of all twins born in England and Wales between 1994 and 1996; and the twins have been followed up into early adulthood. TEDS has assessed the twins' behavioural, cognitive and emotional development from infancy to early adulthood. The present study uses twins' reports of emotional symptoms, social isolation and peer victimisation at 12, 16 and 21 years old (Table 1 provides age estimates for each variable). A total of 15,869 participants (54% females) for whom data were available for at least one measure were included in the phenotypic model. Among these participants, only those for whom zygosity was available were included in the genetic model (*N* = 15,695, including 2,727 complete monozygotic [MZ], 4,837 complete dizygotic [DZ], 138 incomplete MZ and 429 incomplete DZ pairs; Table S1 provides a description of twin pairs for each variable). When twins were about 18 months old, zygosity was determined using a parent‐reported questionnaire of physical similarity (Price et al., 2000). When unclear, zygosity was determined using DNA testing. Participants who were born in extreme perinatal conditions (*N* = 296 twin pairs or 592 individuals), had severe medical conditions (*N* = 95 individuals), or for whom zygosity and/or gender were unknown (*N* = 105 twin pairs or 210 individuals) were excluded.

**Table 1:** Descriptive statistics for emotional symptoms, social isolation and peer victimisation at 12, 16 and 21 years old

	*N*	MZ Pairs		DZ Pairs		Age (*SD*)	Mean (*SD*)	Range	Skew	Kurtosis
		Complete	Incomplete	Complete	Incomplete					
12 years old										
Emotional symptoms	11,681	2,092	33	3,678	59	11.28 (.70)	2.19 (2.06)	0–10	1.02	3.71
Social isolation	11,675	2,090	35	3,674	63	11.28 (.70)	1.15 (1.34)	0–8	1.51	5.73
Peer victimisation	11,729	2,104	23	3,710	28	11.28 (.70)	3.13 (2.88)	0–10	.71	2.45
16 years old										
Emotional symptoms	10,196	1,811	23	3,209	66	16.32 (.68)	2.75 (2.24)	0–10	.75	2.99
Social isolation	10,181	1,804	30	3,202	72	16.32 (.68)	1.47 (1.38)	0–8	1.16	4.44
Peer victimisation	5,236	893	178	1,416	416	16.48 (.27)	2.93 (2.72)	0–10	.75	2.70
21 years old										
Emotional symptoms	9,501	1,271	1,275	2,113	2,454	22.26 (.91)	3.61 (2.70)	0–10	.50	2.29
Social isolation	9,501	1,270	1,276	2,112	2,453	22.26 (.91)	2.08 (1.68)	0–8	.76	3.23
Peer victimisation	8,382	1,155	1,343	1,883	2,556	22.84 (.88)	1.78 (2.21)	0–10	1.26	4.00

Emotional symptoms refer to symptoms of anxiety and depression. Peer victimisation at 16 years old was measured in a separate web survey that was completed by fewer participants for financial reasons. Values are for raw scores (Table S4 shows descriptive statistics for transformed scores). DZ, dizygotic twin; MZ, monozygotic twin; *N*, number of participants; *SD*, standard deviation; Skew, skewness.

### Ethical considerations

Ethical approval for TEDS at the 12‐, 16‐ and 21‐year assessment waves was granted by the King's College London Research Ethics Committee (reference: 05‐Q0706‐228 and PNM 0910 104). TEDS collected informed consent from parents or participants prior to each wave of data collection.

### Measures

#### Emotional symptoms and social isolation

Twins answered questions from the Strengths and Difficulties Questionnaire (SQD; Goodman, 1997) to assess emotional symptoms and social isolation. Participants evaluated how things had been for them over the last three or six months on a three‐point scale (0 = ‘not true’; 1 = ‘quite true’; 2 = ‘very true’). We computed emotional symptoms and social isolation scores by deriving the average of at least three items and multiplying this by the total number of items for each subscale to minimise the impact of missing data.

The emotional symptoms subscale includes five items assessing the extent to which participants experience anxiety and depressive symptoms (e.g., ‘I worry a lot’, ‘I am often unhappy, downhearted, or tearful’). Emotional symptoms scores ranged from 0 to 10. Internal consistency was assessed using ordinal alpha coefficients (Gadermann, Guhn, & Zumbo, 2012) and was satisfactory at 12 (α = .79), 16 (α = .78) and 21 years old (α = .86). Both emotional symptoms and peer problems SDQ subscales show good convergent validity as well as measurement invariance over time (Goodman & Scott, 1999; Speyer, Auyeng, & Murray, 2022; Vugteveen, de Bildt, Theunissen, Reijneveld, & Timmerman, 2021). They both correlate with similar measures from other widely used scales, such as the Child Behaviour Checklist (CBCL) and the Youth Self‐Report (YSR; Achenbach, 1991). The SDQ emotional symptoms subscale is strongly associated with anxiety and depression from the CBCL and YSR, and the SDQ peer problems subscale is strongly correlated with social problems from the CBCL and YSR (Goodman & Scott, 1999; Vugteveen et al., 2021).

We assessed social isolation using four out of the five items of the SDQ peer problems subscale, removing the item ‘Others pick on me or bully me’ to avoid overlap with the peer victimisation measure. The other four items assessed the extent to which participants spend time on their own or are socially accepted by peers (e.g., ‘I am usually on my own. I generally play alone or keep to myself’). Scores ranged from 0 to 8. Internal consistency was satisfactory at 12 (α = .70), 16 (α = .67) and 21 years old (α = .68), as indicated by ordinal alpha coefficients. We also conducted sensitivity analyses using the full five‐item peer problems subscale to see whether removing one item would affect the reliability of the scale. Results suggest that it did not, as they were highly similar to results obtained using the four‐item social isolation measure (see Table S2, Figure S1 and S2).

#### Peer victimisation

We used the self‐reported Multidimensional Peer Victimisation Scale (Mynard & Joseph, 2000) to assess peer victimisation at 12, 16 and 21 years old. Using a three‐point scale (0 = ‘not at all’; 1 = ‘once’; 2 = ‘more than once’), participants answered five items about the extent to which they had experienced acts of physical, verbal and social peer victimisation from other students (at 12 and 16 years) or someone excluding their partner or family (at 21 years), in the past year (e.g., ‘How often has someone refused to talk to you?’). The scale focuses specifically on victimisation by peers at 12 and 16 years, whereas the definition of victimisation is broader at 21 years, encompassing victimisation by individuals from wider social networks (e.g., work colleagues). Despite these differences, the validity and reliability of the Multidimensional Peer Victimisation Scale has been shown to be comparable across developmental periods, including emerging adulthood (Joseph & Stockton, 2018). We computed peer victimisation scores by multiplying the average across at least three items by the total number of items, and scores ranged from 0 to 10. Ordinal alpha coefficients indicated satisfactory internal consistency at 12 (α = .85), 16 (α = .88) and 21 years old (α = .87).

### Statistical analyses

#### Phenotypic analyses

We first transformed all variables using a square root +1 transformation to correct for positive skewness. We conducted descriptive statistics and correlations using RStudio (version 2022.2.2.485; RStudio Team, 2022). The analysis plan was pre‐registered (https://osf.io/sjb5a/?view_only=1ce2374f6e15467ebd2cf626538fbe19). To examine sex differences, we conducted independent samples t‐tests for all variables (see Table S3). We found that females showed higher emotional symptoms than males and that males were more victimised by their peers than females at 12, 16 and 21 years old. We also found that males were more socially isolated than females at 12 years old. To see whether these sex differences were reflected in the patterns of associations between variables, we ran a multi‐group phenotypic cross‐lagged model (see Figure S3A; Figure 2B). We found that a cross‐lagged model in which parameters were freely estimated for females and males showed a better fit than a model in which parameters were constrained to equality across sexes. However, these differences were very small in terms of effect sizes and confidence intervals for most estimates overlapped across females and males. Therefore, we decided not to test a multi‐group genetic cross‐lagged model and to report analyses that controlled for age and sex, as is standard practice for twin model fitting (McGue & Bouchard, 1984).

To examine the bidirectional phenotypic associations between emotional symptoms, social isolation and peer victimisation at all three timepoints, we specified a cross‐lagged panel model using the lavaan package (Rosseel, 2012) to obtain structural equation modelling fit indices (using full‐information maximum likelihood [FIML]). The cross‐lagged model estimated the reciprocal associations between all variables from 12 to 16 years and also from 16 to 21 years. It accounted for the stability across timepoints and pre‐existing relationships between variables, thereby assessing the prediction of incremental changes in variables over time (Adachi & Willoughby, 2015). We then examined the extent to which social isolation at 16 years mediated the bidirectional associations between emotional symptoms and peer victimisation by estimating indirect effects and their 95% confidence intervals (CI) using bootstrapping with 1,000 iterations.

As a sensitivity check, and because twin models are estimated by nesting participants within twin pairs, we ran the model in lavaan using one twin per pair who was randomly chosen (*N* = 7,926) and then using twin pairs in OpenMx (*N* = 8,262 twin pairs; Boker et al., 2011). Estimates were similar across these models, so we report the results of the model using all participants (*N* = 15,869).

#### Genetic analyses

We then examined the genetic and environmental mechanisms underlying the associations between variables using twin modelling. The twin method compares DZ twins, who share on average 50% of their segregating genes, to MZ twins, who share 100% of their genes. Because twins grow up in the same familial environment, contributions from shared environmental factors are expected to increase the similarity between twins. Conversely, non‐shared environmental factors unique to each twin are expected to increase differences between twins. The twin method relies on these assumptions to decompose phenotypic variance and covariance into genetic (A), shared (C) and non‐shared (E) environmental factors (Rijsdijk & Sham, 2002). Non‐shared environmental factors (E) also include measurement error.

We first ran cross‐twin correlations (i.e., between twins of the same family) and univariate twin models to estimate the extent to which A, C and E factors contributed to each variable individually. We estimated cross‐twin cross‐trait correlations between variables (e.g., between trait 1 for twin 1 and trait 2 for twin 2) by running a constrained saturated model, where means and variances were constrained to equality across zygosity and birth order while covariances were constrained to be symmetrical for MZ and DZ twin pairs. We then specified a twin extension of the phenotypic cross‐lagged model, initially described by Burt, McGue, Krueger, and Iacono (2005). Variances (at 12 years) and residual variances (at 16 and 21 years) were decomposed into ACE components. Genetic and environmental correlations were estimated at each timepoint by examining the extent to which genetic and environmental factors account for the covariance between variables using cross‐twin cross‐trait correlations. In addition, gene–environment correlations were examined by focusing on the extent to which genetic factors associated with emotional symptoms overlapped with the other two ‘environmental’ outcomes, that is social isolation and peer victimisation. Such genetic overlap would indicate that a genetic vulnerability for emotional symptoms increases the risk of experiencing interpersonal difficulties, thereby providing evidence for gene–environment correlations. The genetic cross‐lagged model was fitted using FIML and parameter 95% confidence intervals were estimated in OpenMx.

## Results

Table 1 presents descriptive statistics of raw scores (Table S4 presents descriptive statistics for transformed variables), and Table 2 presents phenotypic correlations between variables.

**Table 2:** Correlations between emotional symptoms, social isolation and peer victimisation at 12, 16 and 21 years old

	1.	2.	3.	4.	5.	6.	7.	8.
1. Emotional symptoms 12 years old	1							
2. Social isolation 12 years old	.35 (.33, .37)	1					
3. Peer victimisation 12 years old	.32 (.31, .34)	.27 (.25, 28)	1					
4. Emotional symptoms 16 years old	.34 (.32, .36)	.15 (.13, .17)	.14 (.12, .17)	1				
5. Social isolation 16 years old	.20 (.17, .22)	.30 (.28, .32)	.16 (.14, .18)	.34 (.32, .36)	1			
6. Peer victimisation 16 years old	.18 (.15, .21)	.16 (.13, .19)	.31 (.29, .34)	.23 (.20, .25)	.25 (.22, .28)	1		
7. Emotional symptoms 21 years old	.25 (.23, .28)	.12 (.10, .14)	.14 (.12, .16)	.39 (.37, .41)	.21 (.19, .24)	.18 (.15, .22)	1	
8. Social isolation 21 years old	.17 (.15, .19)	.21 (.19, .23)	.14 (.11, .16)	.20 (.18, .22)	.35 (.33, .37)	.20 (.17, .23)	.38 (.37, .40)	1
9. Peer victimisation 21 years old	.09 (.06, .11)	.10 (.07, .12)	.18 (.15, .20)	.13 (.11, .16)	.11 (.08, .13)	.33 (.30, .36)	.21 (.19, .23)	.12 (.10, .14)

Emotional symptoms refer to symptoms of anxiety and depression.

95% confidence intervals are in parentheses. Correlations were estimated in the constrained saturated model, where means and within‐person correlations were constrained to equality across zygosity, but where correlations between twins were symmetrical across MZ and DZ twins.

### Phenotypic cross‐lagged model

**Figure 1:**
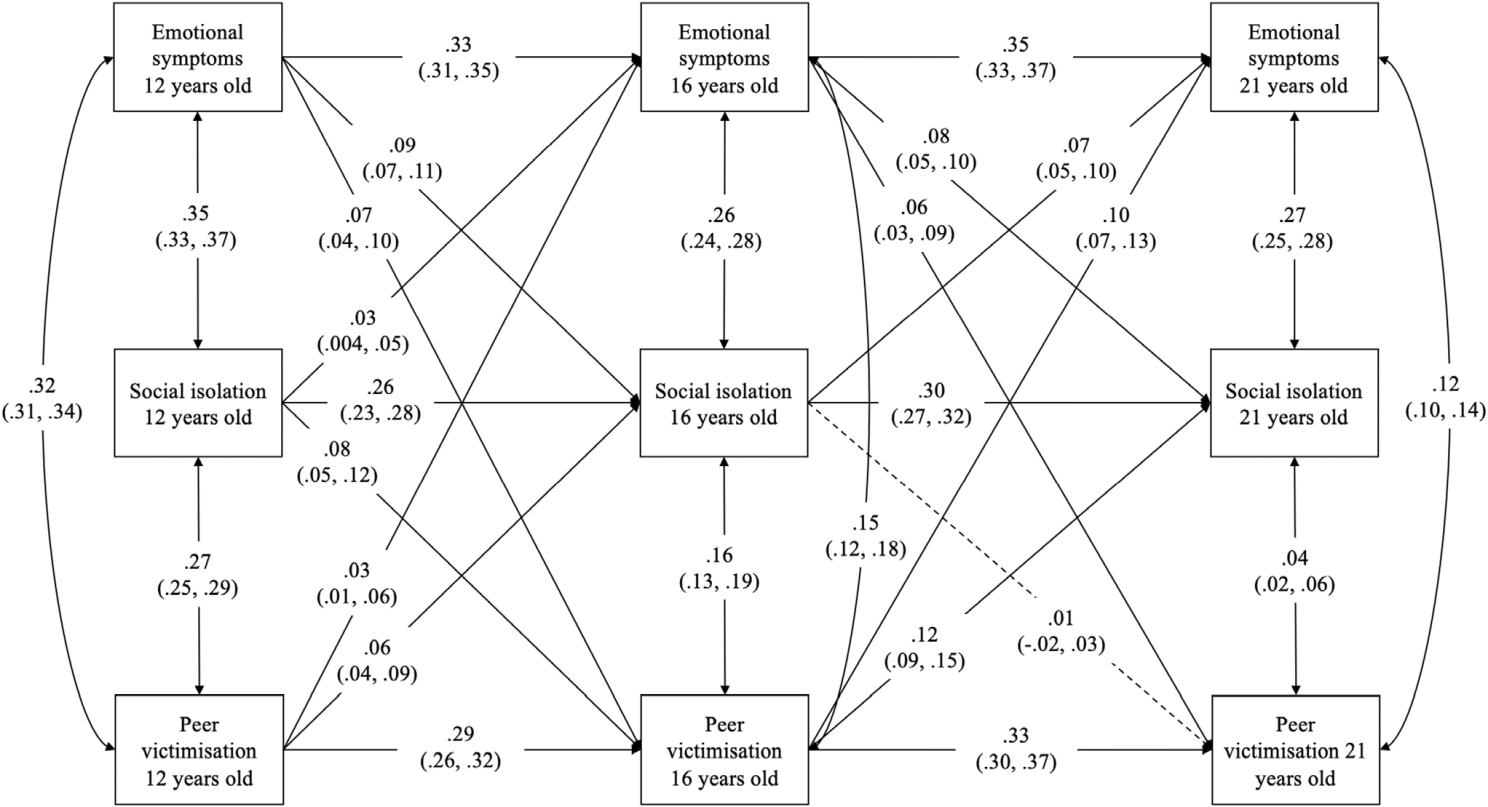
Phenotypic cross‐lagged panel model. *N* = 15,869. Emotional symptoms refer to symptoms of anxiety and depression. Values on single‐headed arrows are standardised partial regression coefficients. These coefficients can be squared to calculate the proportion of variance explained. Values on double‐headed arrows between variables within the same timepoint are correlation coefficients; the correlations at ages 16 and 21 are residuals, indexing the relationship between variables at that age that is not explained by their earlier association. Solid lines indicate statistically significant parameters. The dotted lines indicate non‐significant parameters

We examined the bidirectional associations between emotional symptoms, social isolation and peer victimisation from 12 to 21 years old using a phenotypic cross‐lagged model (Figure 1). Fit indices indicated a good fit of the model: Root Mean Square Error of Approximation (RMSEA) = .04, Comparative Fit Index (CFI) = .98, Tucker‐Lewis Index (TLI) = .91. Cross‐lagged paths were statistically significant for the most part (β = .03 to .12), except for the cross‐lagged path from social isolation at 16 years to peer victimisation at 21 years: β = .01 (95% CI: −.02, .03). These cross‐lagged associations were observed while accounting for the moderate stability in all variables from 12 to 16 years (β = .26 to .33) and from 16 to 21 years (β = .30 to .35). They also accounted for the pre‐existing correlations between variables, which were weak to moderate within each of the timepoints (*r* = .03 to .35).

Overall, the phenotypic cross‐lagged model showed that emotional symptoms at 12 and 16 years were independently and reciprocally associated with both social isolation and peer victimisation at 16 and 21 years, respectively. We then examined whether some of the cross‐lagged paths were stronger than others by comparing models in which the selected parameters were constrained to equality with the unconstrained model in Figure 1 (Table S5 presents these analyses). We found that emotional symptoms equally predicted social isolation and peer victimisation and that conversely, both forms of interpersonal difficulties equally predicted emotional symptoms. In addition, both forms of interpersonal difficulties at 16 years predicted more subsequent emotional symptoms than at 12 years, indicating that social isolation and peer victimisation may carry higher risk for emotional symptoms in mid‐adolescence, compared to early adolescence.

Notably, the model showed that peer victimisation at 12 years indirectly predicted emotional symptoms at 21 years via social isolation at 16 years: β = .004 (95% CI = .003, .007). This indirect effect explained 3% of the correlation between peer victimisation at 12 years and emotional symptoms at 21 years (.004/.141 = .028). However, we did not observe the reverse sequence of associations, as emotional symptoms at 12 years did not predict peer victimisation at 21 years via social isolation: β = .001 (95% CI = −.002, .003).

### Genetic cross‐lagged model

Before testing the full genetic cross‐lagged model, we inspected the cross‐twin correlations and univariate twin models. Cross‐twin correlations suggested significant A (i.e., additive genetic) and E (non‐shared environmental) contributions for most variables, as DZ correlations were about half of MZ correlations (i.e., indicating A influences) and MZ correlations were less than 1 (i.e., indicating E influences). Univariate twin models confirmed that most variables were accounted for by A and E influences (Table 3, see Table S6 for fit comparisons of univariate twin models). We then specified the genetic cross‐lagged model informed by cross‐twin cross‐trait correlations (Table S7; Burt et al., 2005). We first tested an ACE model and then dropped C parameters that were non‐significant or close to 0, one at a time. We compared nested models using likelihood ratio tests. The final model included AE factors for most variables, except for victimisation at 12 years old, which had a significant C component. The genetic model had a better fit than the phenotypic cross‐lagged model, suggesting that adding A and E components improved model fit (likelihood ratio test, *p* < .001; Akaike's Information Criterion [AIC] = 237,463.60 and 234,391.90 for the phenotypic and genetic models, respectively [lower AIC values suggesting better fit]).

**Table 3:** Cross‐twin correlations and univariate estimates for emotional symptoms, social isolation and peer victimisation at 12, 16 and 21 years old

	*r*MZ	*r*DZ	A	C	E
12 years old					
Emotional symptoms	.40 (.36, .44)	.20 (.17, .23)	.40 (.37, .43)	–	.60 (.57, .63)
Social isolation	.37 (.33, .41)	.21 (.18, .24)	.39 (.36, .42)	–	.61 (.58, .64)
Peer victimisation	.57 (.54, .60)	.33 (.30, .36)	.45 (.37, .52)	.11 (.05, .18)	.44 (.41, .47)
16 years old					
Emotional symptoms	.42 (.38, .46)	.16 (.13, .20)	.40 (.36, .43)	–	.60 (.57, .64)
Social isolation	.41 (.37, .45)	.20 (.17, .23)	.41 (.38, .44)	–	.59 (.56, .62)
Peer victimisation	.46 (.41, .51)	.20 (.15, .25)	.45 (.41, .50)	–	.55 (.50, .59)
21 years old					
Emotional symptoms	.34 (.30, .39)	.19 (.15, .23)	.35 (.31, .39)	–	.65 (.61, .69)
Social isolation	.39 (.35, .43)	.19 (.16, .23)	.39 (.35, .42)	–	.61 (.58, .65)
Peer victimisation	.29 (.24, .34)	.14 (.10, .18)	.29 (.25, .34)	–	.71 (.66, .75)

Emotional symptoms refer to symptoms of anxiety and depression. 95% confidence intervals are in parentheses. A, additive genetic factors; C, shared environmental factors; E, non‐shared environmental factors; *r*DZ, cross‐twin correlations for dizygotic twins; *r*MZ, cross‐twin correlations for monozygotic twins.

**Figure 2:**
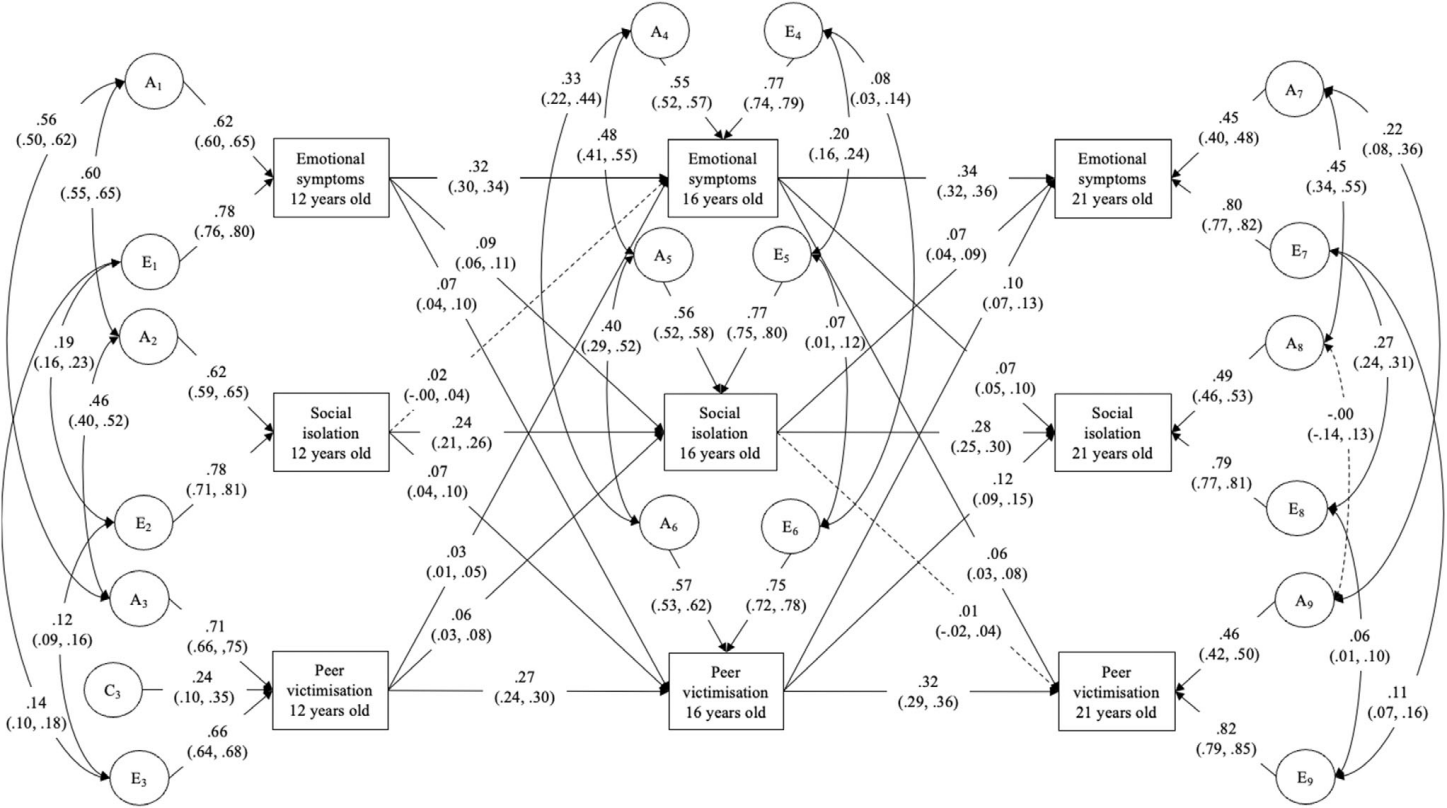
Genetic cross‐lagged panel model. *N* = 15,695, including 2,727 complete MZ, 4,837 complete DZ, 138 incomplete MZ and 429 incomplete DZ pairs. Emotional symptoms refer to symptoms of anxiety and depression. A, additive genetic factors; C, shared‐environmental factors; E, environmental factors. Values on single‐headed arrows are standardised path estimates; values on double‐headed arrows are correlation coefficients. Solid lines indicate statistically significant parameters. The dotted lines indicate non‐significant parameters. Proportion of variance explained by A and E components can be estimated by squaring their respective path estimates

**Figure 3:**
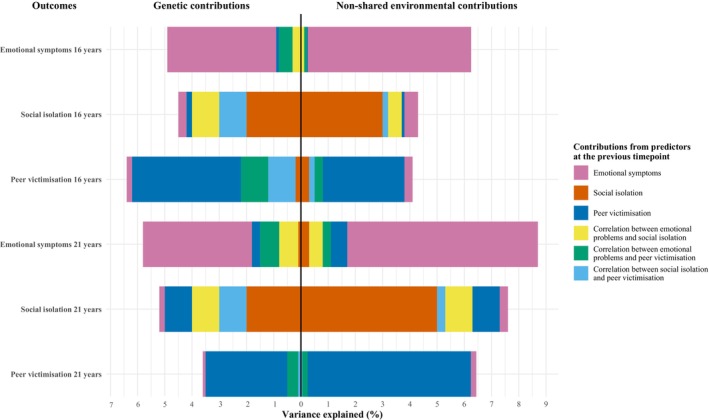
Proportion of variance explained in outcomes from the genetic cross‐lagged model presented in Figure 2. Emotional symptoms refer to anxiety and depression symptoms. Each bar represents the variance in outcome variables explained by genetic and non‐shared environmental contributions from previous variables (i.e., contributions transmitted over time). For example, the top bar shows the genetic and non‐shared environmental contributions to emotional symptoms at 16 years old from variables at 12 years old. The left‐hand side of the bar shows the genetic contributions, and the right‐hand side shows the non‐shared environmental contributions. Each colour represents the predicting variable from which these contributions originate. The pink section on the left‐hand side of the bar shows, for example, that approximately 4% of the variance in emotional symptoms at 16 years old is explained by genetic contributions unique to emotional symptoms at 12 years old (the exact estimate is provided in Table 4). By comparison, the pink section on the right‐hand side shows that approximately 6% of the variance in emotional symptoms at 16 years old is explained by non‐shared environmental factors contributions unique to emotional symptoms at 12 years old.

The genetic model, presented in Figure 2, shows that A and E estimates for emotional symptoms and social isolation at 12 years were similar, with E accounting for a larger proportion of variance (61%) than A (39%). Findings were somewhat different for peer victimisation at 12 years, with A (50%) and E (44%) explaining comparable proportions of variance (C, or non‐shared environmental factors, explained 6% of the variance in peer victimisation at 12 years old). At 16 and 21 years, most of the variance was accounted for by new A (20% to 32%) and E (56% to 67%) components, indicating time‐specific genetic and non‐shared environmental contributions. The remaining part of the variance in outcome variables was accounted for by previous A and E influences transmitted via stability (2% to 7%) and cross‐lagged paths (.01% to 1%). Total transmitted contributions accounted for 9 to 13% of the variance in outcomes at 16 and 21 years old, indicating that outcome variables were only partially explained by variables at previous timepoints. These contributions from previous timepoints, along with their genetic and environmental components, are presented in Figure 3 and Table 4. Overall, findings from the genetic cross‐lagged model indicate that, for the most part, non‐shared environmental influences accounted for the largest proportion of variability in emotional symptoms, social isolation and peer victimisation at all timepoints and that most of the variance in outcome variables at 16 and 21 years was explained by new aetiological influences emerging over time.

**Table 4:** Proportions of genetic and environmental variance accounted for in emotional symptoms, social isolation and peer victimisation at 16 and 21 years old

	A	E	Total phenotypic variance explained
*Contributions to emotional symptoms at age 16*			
Unique effects of			
Stability from emotional symptoms age 12	.04 (11%)	.06 (9%)	.10
Cross‐lagged from social isolation age 12	.0001 (.04%)	.0002 (.03%)	.0003
Cross‐lagged from peer victimisation age 12	.001 (.1%)	.0003 (.1%)	.001
Common effects of pre‐existing correlation with			
Social isolation age 12	.003 (1%)	.001 (.2%)	.004
Peer victimisation age 12	.005 (1%)	.001 (.2%)	.006
Residual factors	.30 (86%)	.59 (90%)	.89
Total AE components	.35 (100%)	.65 (100%)	1.00
*Contributions to social isolation at age 16*			
Unique effects of			
Stability from social isolation age 12	.02 (6%)	.03 (5%)	.06
Cross‐lagged from emotional symptoms age 12	.003 (1%)	.005 (1%)	.01
Cross‐lagged from peer victimisation age 12	.002 (1%)	.001 (.2%)	.003
Common effects of pre‐existing correlation with			
Emotional symptoms age 12	.01 (3%)	.005 (1%)	.01
Peer victimisation age 12	.01 (2%)	.002 (.3%)	.01
Residual factors	.31 (88%)	.60 (93%)	.91
Total AE components	.35 (100%)	.65 (100%)	1.00
*Contributions to peer victimisation at age 16*			
Unique effects of			
Stability from peer victimisation age 12	.04 (9%)	.03 (5%)	.07
Cross‐lagged from emotional symptoms age 12	.002 (1%)	.003 (1%)	.005
Cross‐lagged from social isolation age 12	.002 (1%)	.003 (1%)	.005
Common effects of pre‐existing correlation with			
Emotional symptoms age 12	.01 (2%)	.003 (.4%)	.01
Social isolation age 12	.01 (2%)	.002 (.4%)	.01
Residual factors	.33 (85%)	.56 (93%)	.89
Total AE components	.39 (100%)	.61 (100%)	.99^a^
*Contributions to emotional symptoms at age 21*			
Unique effects of			
Stability from emotional symptoms age 16	.0351 (14%)	.0680 (9%)	.1031
Cross‐lagged from social isolation age 16	.0013 (1%)	.0025 (.4%)	.0038
Cross‐lagged from peer victimisation age 16	.0034 (1%)	.0059 (1%)	.0093
Common effects of pre‐existing correlation with			
Social isolation age 16	.0065 (3%)	.0053 (1%)	.0117
Peer victimisation age 16	.0073 (3%)	.0032 (.4%)	.0105
Residual factors	.1980 (79%)	.6376 (88%)	.8356
Total AE components (excluding those at 12 years)	.2516 (100%)	.7225 (100%)	.9741
*Contributions to social isolation at age 21*			
Unique effects of			
Stability from social isolation age 16	.02 (8%)	.05 (7%)	.07
Cross‐lagged from emotional symptoms age 16	.002 (1%)	.003 (1%)	.01
Cross‐lagged from peer victimisation age 16	.01 (2%)	.01 (1%)	.01
Common effects of pre‐existing correlation with			
Emotional symptoms age 16	.01 (2%)	.01 (1%)	.01
Peer victimisation age 16	.01 (3%)	.003 (.4%)	.01
Residual factors	.24 (84%)	.63 (91%)	.87
Total AE components (excluding those at 12 years)	.29 (100%)	.69 (100%)	.98
*Contributions to peer victimisation at age 21*			
Unique effects of			
Stability from peer victimisation age 16	.03 (14%)	.06 (8%)	.09
Cross‐lagged from emotional symptoms age 16	.001 (.4%)	.002 (.3%)	.003
Cross‐lagged from social isolation age 16	.00003 (.01%)	.0001 (.01%)	.0001
Common effects of pre‐existing correlation with			
Emotional symptoms age 16	.004 (2%)	.002 (.2%)	.01
Social isolation age 16	.001 (.3%)	.0003 (.04%)	.001
Residual factors	.21 (83%)	.67 (91%)	.88
Total AE components (excluding those at 12 years)	.25 (100%)	.74 (100%)	.98

Unique A (i.e., additive genetic) and E (non‐shared environmental) effects transmitted via stability and cross‐lagged paths were calculated by squaring the product of the phenotypic path by the A or E path on the previous corresponding variable. For example, the A effects transmitted via stability of emotional symptoms from 12 to 16 years old were calculated based on estimates shown in Figure 2: (.32*.62)^2^ = .04. Common A and E effects of pre‐existing correlations were computed by multiplying by two the product of all paths connecting two variables via prior A or E influences. For example, the common A effects on emotional symptoms at 16 years due to the pre‐existing correlation between emotional symptoms and social isolation were based on estimates shown in Figure 2: 2*(.32*.62*.60*.62*.02) = .003.

To avoid overcrowding the table, contributions to outcomes at age 21 years only included effects from A and E factors at age 16, which explains why the total phenotypic variance at age 21 years is smaller than 1.

In parentheses are the percentages of contributions to total A and E influences of each variable.

^a^ Proportions do not add up to one because peer victimisation at 12 years old had a C component.

Genetic and environmental correlations were mostly significant, indicating that emotional symptoms, social isolation and peer victimisation shared both genetic and environmental risks at each timepoint. Genetic correlations were moderate at 12 years and were weaker at later timepoints (*r*G at 12 years = .46 to .60; *r*G at 16 years = .33 to .48; *r*G at 21 years = .00 to .45). These findings suggest that gene–environment correlations partially account for the relationships between emotional symptoms and both social isolation and peer victimisation. Non‐shared environmental correlations were weak but comparable at each timepoint (*r*E at 12 years = .12 to .19; *r*E at 16 years = .07 to .20; *r*E at 21 years = .06 and .27), indicating that individual‐specific environmental pathways modestly accounted for the associations between interpersonal difficulties and emotional symptoms.

## Discussion

The present study is the first to use a genetically sensitive design to document the reciprocal associations between emotional symptoms (i.e., anxiety and depressive symptoms) and two different forms of interpersonal difficulties, social isolation and peer victimisation, over an extended period during adolescence and early adulthood. First, we found that emotional symptoms predicted increases in social isolation and peer victimisation at later timepoints, and that in turn, social isolation and peer victimisation independently contributed to emotional symptoms over time. We also found a significant longitudinal mediation where social isolation in mid‐adolescence partly explained the long‐term link between peer victimisation at 12 years and emotional problems at 21 years. Second, twin analyses showed that, for the most part, non‐shared environmental influences mostly explained individual differences in all variables during adolescence and early adulthood and that both gene–environment and non‐shared environmental correlations accounted for their associations at each timepoint.

Our findings align with prior studies supporting a transactional model of reciprocal influences between emotional symptoms and negative peer interactions (e.g., Smith et al., 2022), but also extend them by including another aspect of interpersonal difficulties. Prior research has mainly focused on the links between emotional symptoms and peer victimisation without considering that adolescents experiencing anxiety and depressive symptoms may also experience other, maybe less obvious, types of interpersonal difficulties such as social isolation. Our study shows that social isolation carries as much risk as peer victimisation when it comes to exacerbating emotional symptoms during adolescence. As peer relationships become more salient during adolescence, young individuals who show persistent difficulties with social relationships may be viewed as deviating from the peer group norms (Brown & Larson, 2009; Oh et al., 2008; Rubin, Bukowski, & Parker, 2006). This may be the case for emotionally vulnerable adolescents, who often struggle with anxiety in social situations (Nilsen, Karevold, Roysamb, Gustavson, & Mathiesen, 2013; Pickard, Rijsdijk, Happé, & Mandy, 2017). Consequently, these adolescents may miss out on opportunities to create meaningful relationships and gain confidence in their social abilities, which may ultimately exacerbate emotional problems over time (Rubin et al., 2009). Our results also suggest that social isolation and peer victimisation have a greater impact on long‐term emotional symptoms if experienced at 16 years, compared to earlier. Experiencing interpersonal difficulties in mid‐adolescence may affect young people's capacity to navigate the transition to early adulthood, a period marking important career and educational choices. As a result, it may be harder for young individuals who lack the social skills and confidence necessary to establish a supportive social network to maintain emotional well‐being during this transition (Nikitin & Freund, 2008).

In addition, our results provide some evidence that social isolation in late adolescence may be an intermediate pathway for the relationship between peer victimisation and emotional symptoms. Peer victimisation in early adolescence predicted later emotional symptoms in emerging adulthood via social isolation, suggesting that adolescents who experience peer victimisation during the first years of secondary school may develop a tendency to withdraw from social interactions (Barzeva et al., 2020; Oh et al., 2008). In turn, being socially isolated reduces opportunities for socialising and establishing new relationships during the transition to emerging adulthood, thereby leading to more distress and emotional symptoms over time (Oh et al., 2008; Rubin et al., 2006, 2009). However, social isolation did not significantly explain the link between emotional symptoms in early adolescence and peer victimisation in emerging adulthood, as social isolation in mid‐adolescence did not significantly predict later peer victimisation. This could be explained by the increased freedom to decide whether and with whom to interact socially in young adulthood (e.g., at work or university; Nikitin & Freund, 2008). Young adults may be in a better position to avoid social situations conducive to peer victimisation. Peer victimisation may also be less common once adolescents finish secondary school and are no longer exposed to their peers every day. Therefore, although social isolation in mid‐adolescence was associated with later increases in emotional symptoms, our findings suggest that being socially isolated at 16 years may carry less risk for peer victimisation in emerging adulthood.

We further found that non‐shared environmental influences accounted for most of individual differences in emotional symptoms, social isolation and peer victimisation and that the magnitude of these influences was consistent across timepoints. Because the cross‐lagged model accounted for the stability and prior relationships between variables, non‐shared environmental contributions at 16 and 21 years old can be interpreted as explaining incremental change in variables over time. As such, this finding is consistent with longitudinal twin studies showing that change in emotional and social problems is primarily accounted for by environmental influences (Brendgen et al., 2022; Matthews et al., 2022; Nivard et al., 2015). Some of these non‐shared environmental contributions could include risk factors with a broader impact on both emotional and social domains such as negative relationships with parents and peers (Kaufman, Kretschmer, Huitsing, & Veenstra, 2019).

Our findings further suggest that both gene–environment non‐shared environmental correlations partially accounted for the associations between emotional symptoms, social isolation and peer victimisation at each timepoint. This finding is in line with studies showing genetic and environmental overlap between emotional symptoms and negative peer experiences but also extends them by providing evidence of these etiological processes at different periods during adolescence and early adulthood (Brendgen et al., 2013, 2022; Matthews et al., 2016; Schaefer et al., 2018; Silberg et al., 2016; Smith et al., 2021). Whereas gene–environment correlations suggest that the risk of experiencing interpersonal difficulties may be traced back to adolescents' genetic predisposition to emotional symptoms, non‐shared environmental correlations point to the possibility that the relationships between emotional symptoms and interpersonal difficulties are partly due to direct phenotypic pathways (i.e., unconfounded by genetic and familial vulnerabilities). Interestingly, the gene–environment correlations were larger at 12 years than at later timepoints, while the magnitude of non‐shared environmental correlations was consistent across timepoints. This could be explained by the fact that stability in behavioural traits, which is accounted for in the cross‐lagged model, is substantially accounted for by genetic factors (Nivard et al., 2015). Nonetheless, this means that new etiological processes emerge over time, perhaps suggesting that the genetic and non‐shared environmental factors responsible for the relationships between emotional symptoms and interpersonal difficulties are not the same at 12, 16 and 21 years.

Our results must be interpreted while considering some limitations. First, the cross‐lagged model does not disentangle between‐ from within‐person effects and cannot be used to answer causal inference hypotheses (Hamaker, Kuiper, & Grasman, 2015). Our findings could either reflect between‐person processes, where adolescents who manifest more emotional symptoms than their peers are also more victimised, or within‐person processes, where adolescents' fluctuations in emotional symptoms lead to more peer victimisation than what they usually experience. We could have examined these within‐person associations more specifically by testing a random‐intercept cross‐lagged model (Hamaker et al., 2015). However, because we aimed to understand why some adolescents were at higher risk for emotional symptoms than others and examine the risk factors associated with the persistence of emotional symptoms, the cross‐lagged panel model was more appropriate. The cross‐lagged panel model was also suitable to twin decomposition, as shown in previous studies using the same twin model (e.g., Burt et al., 2005), and examining the genetic and environmental associations between variables was a central aim of our study. Further methodological work would have been required to apply the twin method to a random‐intercept cross‐lagged model, as this has never been done before. Such methodological work was beyond the scope of the present study. Second, some of the variances in self‐reported measures may be due to self‐perception biases and may be responsible for inflating within‐person correlations between variables (i.e., common method variance) or increasing non‐shared environmental variance in twin analyses. Third, assessments were separated by a large time interval (i.e., 4–5 years) and this may have affected our capacity to identify the specific developmental periods at which risks were most important.

## Conclusions

In summary, our study shows that the mechanisms underlying emotional symptoms during adolescence involve reciprocal associations with social isolation and peer victimisation and that both genetic and environmental influences play a role in these mechanisms. If replicated, our findings highlight the necessity to intervene early in adolescence and to pay attention to the different types of interpersonal difficulties that emotionally vulnerable adolescents may experience, including social isolation and peer victimisation, to prevent their exacerbation over time.

## Supporting information


**Table S1.** Number of complete and incomplete twin pairs for each variable.
**Table S2.** Sensitivity correlations between emotional symptoms, peer problems and peer victimisation at 12, 16 and 21 years old.
**Table S3.** Independent sample t‐tests to examine sex differences.
**Table S4.** Descriptive statistics for transformed variables at 12, 16 and 21 years old.
**Table S5.** Comparison of phenotypic cross‐lagged models.
**Table S6.** Fit comparisons for univariate twin models.
**Table S7.** Cross‐twin cross‐trait correlations,
**Figure S1.** Phenotypic cross‐lagged model using the full peer problems scale (sensitivity analysis).
**Figure S2.** Genetic cross‐lagged model using the full peer problems scale (sensitivity analysis).
**Figure S3.** Multi‐group phenotypic cross‐lagged model (sex differences).
