# Supplementary material for: A cross‐lagged twin study of emotional symptoms, social isolation and peer victimisation from early adolescence to emerging adulthood
Source: J Child Psychol Psychiatry. 2023 Jun 6;64(11):1569–82. doi: 10.1111/jcpp.13847 (PMC7615178; doi:10.1111/jcpp.13847)
Supplement: Supplementary file 1 — Table S1. Number of complete and incomplete twin pairs for each variable. Table S2. Sensitivity correlations between emotional symptoms, peer problems and peer victimisation at 12, 16 and 21 years old. Table S3. Independent sample t‐tests to examine sex differences. Table S4. Descriptive statistics for transformed variables at 12, 16 and 21 years old. Table S5. Comparison of phenotypic cross‐lagged models. Table S6. Fit comparisons for univariate twin models. Table S7. Cross‐twin cross‐trait correlations, Figure S1. Phenotypic cross‐lagged model using the full peer problems scale (sensitivity analysis). Figure S2. Genetic cross‐lagged model using the full peer problems scale (sensitivity analysis). Figure S3. Multi‐group phenotypic cross‐lagged model (sex differences). [file JCPP-64-1569-s001.docx]

**Supporting Information**

| Table S1. Number of Complete and Incomplete Twin Pairs for Each Variable | | | | | | | | | | |  |  |
| --- | --- | --- | --- | --- | --- | --- | --- | --- | --- | --- | --- | --- |
|  | MZ Female Pairs | | MZ Male Pairs | | DZ Female Pairs | | DZ Male Pairs | | DZ Opposite-Sex Pairs | | Total | |
|  | Full | Inc. | Full | Inc. | Full | Inc. | Full | Inc. | Full | Inc. | Full | Inc. |
| ES 12 | 1,140 | 18 | 952 | 15 | 997 | 11 | 874 | 12 | 1,807 | 36 | 5,770 | 92 |
| SI 12 | 1,140 | 18 | 950 | 17 | 998 | 10 | 871 | 15 | 1,805 | 38 | 5,764 | 98 |
| PV 12 | 1,148 | 12 | 956 | 11 | 1,008 | 3 | 878 | 7 | 1,824 | 18 | 5,814 | 51 |
| ES 16 | 1,065 | 9 | 746 | 14 | 924 | 14 | 694 | 12 | 1,591 | 40 | 5,020 | 89 |
| SI 16 | 1,059 | 15 | 745 | 15 | 921 | 17 | 693 | 13 | 1,588 | 42 | 5,006 | 102 |
| PV 16 | 561 | 90 | 332 | 88 | 446 | 87 | 304 | 87 | 666 | 242 | 2,309 | 594 |
| ES 21 | 829 | 615 | 442 | 660 | 686 | 591 | 392 | 633 | 1,035 | 1,230 | 3,384 | 3,729 |
| SI 21 | 829 | 615 | 441 | 661 | 686 | 591 | 392 | 632 | 1,034 | 1,230 | 3,382 | 3,729 |
| PV 21 | 767 | 643 | 388 | 700 | 642 | 612 | 345 | 653 | 896 | 1,291 | 3,038 | 3,899 |
| Abbreviations: MZ = monozygotic; DZ = dizygotic; Inc. = incomplete; ES = emotional symptoms; SI = social isolation; PV = peer victimisation; 12 = 12 years old; 16 = 16 years old; 21 = 21 years old. | | | | | | | | | | | | |

| Table S2. Sensitivity Correlations Between Emotional Symptoms, Peer Problems, and Peer Victimisation at 12, 16, and 21 Years Old | | | | | | | | |
| --- | --- | --- | --- | --- | --- | --- | --- | --- |
|  | 1. | 2. | 3. | 4. | 5. | 6. | 7. | 8. |
| 1. Emotional Symptoms 12 Years | 1 |  |  |  |  |  |  |  |
| 2. Peer Problems 12 Years | .39  (.36, .41) | 1 |  |  |  |  |  |  |
| 3. Peer Victimisation 12 Years | .32  (.31, .34) | .35  (.33, .37) | 1 |  |  |  |  |  |
| 4. Emotional Symptoms 16 Years | .34  (.32, .36) | .17  (.14, .20) | .14  (.12, .17) | 1 |  |  |  |  |
| 5. Peer Problems 16 Years | .22  (.19, .25) | .33  (.30, .36) | .17  (.14, .20) | .36  (.34, .39) | 1 |  |  |  |
| 6. Peer Victimisation 16 Years | .18  (.15, .21) | .19  (.15, .23) | .31  (.29, .34) | .23  (.20, .25) | .28  (.24, .32) | 1 |  |  |
| 7. Emotional Symptoms 21 Years | .25  (.23, .28) | .16  (.12, .19) | .14  (.12, .16) | .39  (.37, .41) | .24  (.21, .27) | .18  (.15, .22) | 1 |  |
| 8. Peer Problems 21 Years | .16  (.13, .19) | .22  (.18, .25) | .10  (.07, .13) | .22  (.19, .25) | .35  (.32, .38) | .18  (.13, .23) | .42  (.39, .44) | 1 |
| 9. Peer Victimisation 21 Years | .09  (.06, .11) | .11  (.08, .15) | .18  (.15, .20) | .13  (.11, .16) | .12  (.08, .15) | .33  (.30, .36) | .21  (.19, .23) | .15  (.11, .18) |
| *Note*. 95% confidence intervals are in parentheses. Correlations were estimated in the constrained saturated model, where means and within-person correlations were constrained to equality across zygosity, but where correlations between twins were symmetrical for MZ and DZ twins. Internal consistency of the full five-item peer problems scale, as indicated by ordinal alpha coefficients, was .68, .73, and .71 at 12, 16, and 21 years old respectively. | | | | | | | | |

| Table S3. Independent Sample T-Tests to Examine Sex Differences | | | | | |
| --- | --- | --- | --- | --- | --- |
|  | Mean Females (SD) | Mean Males (SD) | *t* | df | *p*-value |
| *12 Years Old* | | | | | |
| **Emotional Symptoms** | **2.39 (2.12)** | **1.97 (1.97)** | **11.03** | **11679** | **< .001** |
| **Social Isolation** | **1.07 (1.30)** | **1.25 (1.37)** | **-7.20** | **11673** | **< .001** |
| **Peer Victimisation** | **2.83 (2.77)** | **3.47 (2.97)** | **-12.01** | **11727** | **< .001** |
| *16 Years Old* | | | | | |
| **Emotional Symptoms** | **3.40 (2.31)** | **1.94 (1.86)** | **34.57** | **10194** | **< .001** |
| Social Isolation | 1.44 (1.37) | 1.51 (1.39) | -2.65 | 10179 | .008 |
| **Peer Victimisation** | **2.64 (2.66)** | **3.35 (2.75)** | **-9.39** | **5234** | **< .001** |
| *21 Years Old* | | | | | |
| **Emotional Symptoms** | **4.12 (2.71)** | **2.77 (2.45)** | **24.35** | **9499** | **< .001** |
| Social Isolation | 2.08 (1.71) | 2.07 (1.62) | .19 | 9499 | .85 |
| **Peer Victimisation** | **1.60 (2.13)** | **2.08 (2.31)** | **-9.51** | **8380** | **< .001** |
| *Note*. Independent-sample t-tests were performed on raw variables (before square root transformations). Emotional symptoms and peer victimisation scores ranged from 0 to 10. Social isolation scores ranged from 0 to 8. In bold are statistically significant t-tests, after adjusting for multiple tests using the Bonferroni correction (adjusting for 9 tests, .05/9 = .006). | | | | | |

| Table S4. Descriptive Statistics for Transformed Variables at 12, 16, and 21 Years Old | | | | | |
| --- | --- | --- | --- | --- | --- |
|  | N | Mean (SD) | Range | Skewness | Kurtosis |
| *12 Years Old* | | | |  |  |
| Emotional Symptoms | 11 681 | .00 (1.00) | -1.39-3.10 | .43 | -.60 |
| Social Isolation | 11 675 | .00 (1.00) | -1.09-3.90 | .85 | .23 |
| Peer Victimisation | 11 729 | .00 (1.00) | -1.64-2.37 | .26 | -1.09 |
| *16 Years Old* | | | |  |  |
| Emotional Symptoms | 10 196 | .00 (1.00) | -1.86-3.04 | .20 | -.78 |
| Social Isolation | 10 181 | .00 (1.00) | -1.41-3.58 | .54 | -.21 |
| Peer Victimisation | 5 236 | .00 (1.00) | -1.47-2.28 | .25 | -1.03 |
| *21 Years Old* | | | |  |  |
| Emotional Symptoms | 9 501 | .00 (1.00) | -1.92-2.44 | .02 | -.97 |
| Social Isolation | 9 501 | .00 (1.00) | -1.49-2.77 | .20 | -.66 |
| Peer Victimisation | 8 382 | .00 (1.00) | -1.22-3.12 | .76 | -.54 |
| *Note*. We performed a square root + 1 transformation on all variables. Variables were regressed on age, sex, and age*sex, and we saved z-standardised residuals and used them in the main analyses. Abbreviations: N = number of participants; SD = standard deviation. | | | | | |

| Table S5. Comparison of Phenotypic Cross-Lagged Models | | | | | |
| --- | --- | --- | --- | --- | --- |
|  | -2LL | Δ -2LL | df | Δ df | *p* |
| *Constraining Cross-Lagged Paths from Emotional Symptoms to Social Isolation and Peer Victimisation to Equality (One Equality Constraint for the Two Paths from 12 to 16, and Another for the Two Paths from 16 to 21 Years)* | | | | | |
| Constrained Model | 238,958.60 |  |  |  |  |
| Unconstrained Model | 238,955.79 | 2.81 | 9 | 2 | .24 |
| *Constraining Cross-Lagged Paths from Social Isolation and Peer Victimisation to Emotional Symptoms to Equality (One Equality Constraint for the Two Paths from 12 to 16, and Another for the Two Paths from 16 to 21 Years)* | | | | | |
| Constrained Model | 238,957.38 |  | 11 |  |  |
| Unconstrained Model | 238,955.79 | 1.59 | 9 | 2 | .45 |
| *Constraining All Cross-Lagged Paths from Social Isolation to Emotional Symptoms to Equality* | | | | | |
| Constrained Model | 238,963.41 |  | 10 |  |  |
| Unconstrained Model | 238,955.79 | 7.62 | 9 | 1 | .01 |
| *Constraining All Cross-Lagged Paths from Peer Victimisation to Emotional Symptoms to Equality* | | | | | |
| Constrained Model | 238,967.08 |  | 10 |  |  |
| Unconstrained Model | 238,955.79 | 11.29 | 9 | 1 | .00 |
| *Note*. *p*-values were estimated with chi-square tests. Abbreviations: LL = log likelihood; df = degrees of freedom. | | | | | |

| Table S6. Fit Comparisons for Univariate Twin Models | | | | | | | |
| --- | --- | --- | --- | --- | --- | --- | --- |
| Base Model | Comparison Model | -2LL | df | AIC | Δ -2LL | Δ df | *p* |
| *Emotional Symptoms 12 Years Old* | | | | | | | |
| Saturated | - | 32488.41 | 11622 | 32508.41 | N/A | N/A | N/A |
| Saturated | ACE | 32493.36 | 11628 | 32501.36 | 4.95 | 6 | .55 |
| ACE | AE | 32493.40 | 11629 | 32499.40 | 0.04 | 1 | .85 |
| *Social Isolation 12 Years Old* | | | | | | | |
| Saturated | - | 32521.56 | 11616 | 32541.56 | N/A | N/A | N/A |
| Saturated | ACE | 32521.56 | 11622 | 32537.17 | 7.61 | 6 | .27 |
| ACE | AE | 32530.63 | 11623 | 32536.63 | 1.46 | 1 | .23 |
| *Peer Victimisation 12 Years Old* | | | | | | | |
| Saturated | - | 31864.87 | 11669 | 31884.87 | N/A | N/A | N/A |
| Saturated | ACE | 31864.87 | 11675 | 31884.87 | 7.27 | 6 | 0.30 |
| ACE | AE | 31885.47 | 11676 | 31891.47 | 13.33 | 1 | 0.00 |
| *Emotional Symptoms 16 Years Old* | | | | | | | |
| Saturated | - | 28308.28 | 10119 | 28328.28 | N/A | N/A | N/A |
| Saturated | ACE | 28319.34 | 10125 | 28327.34 | 11.06 | 6 | .09 |
| ACE | AE | 28319.34 | 10126 | 28325.34 | .00 | 1 | 1 |
| *Social Isolation 16 Years Old* | | | | | | | |
| Saturated | - | 28218.13 | 10104 | 28238.13 | N/A | N/A | N/A |
| Saturated | ACE | 28225.59 | 10110 | 28233.59 | 7.46 | 6 | .28 |
| ACE | AE | 28225.59 | 10111 | 28231.59 | .00 | 1 | 1 |
| *Peer Victimisation 16 Years Old* | | | | | | | |
| Saturated | - | 14509.36 | 5202 | 14529.36 | N/A | N/A | N/A |
| Saturated | ACE | 14514.19 | 5208 | 14522.19 | 4.82 | 6 | .57 |
| ACE | AE | 14514.19 | 5209 | 14520.19 | .00 | 1 | 1 |
| *Emotional Symptoms 21 Years Old* | | | | | | | |
| Saturated | - | 26428.12 | 9401 | 26448.12 | N/A | N/A | N/A |
| Saturated | ACE | 26428.12 | 9407 | 26440.94 | 4.82 | 6 | .57 |
| ACE | AE | 26433.27 | 9408 | 26439.27 | .34 | 1 | .56 |
| *Social Isolation 21 Years Old* | | | | | | | |
| Saturated | - | 26354.60 | 9401 | 26374.60 | N/A | N/A | N/A |
| Saturated | ACE | 26356.08 | 9407 | 26364.08 | 1.48 | 6 | .96 |
| ACE | AE | 26356.15 | 9408 | 26362.15 | 0.07 | 1 | .79 |
| *Peer Victimisation 21 Years Old* | | | | | | | |
| Saturated | - | 23387.10 | 8289 | 23407.10 | N/A | N/A | N/A |
| Saturated | ACE | 23400.59 | 8295 | 23408.59 | 13.49 | 6 | .04 |
| ACE | AE | 23400.59 | 8296 | 23406.59 | .00 | 1 | 1 |
| *Note.* Abbreviations: -2LL = minus twice the log likelihood; df = degrees of freedom; AIC = Akaike’s information criterion; A = additive genetic factors; C= shared environmental factors; E = non-shared environmental factors. | | | | | | | |

| Table S7. Cross-Twin Cross-Trait Correlations | | | | | | | | | | |
| --- | --- | --- | --- | --- | --- | --- | --- | --- | --- | --- |
|  |  | MZ Twin 2 | | | | | | | | |
|  |  | ES 12 | SI 12 | PV 12 | ES 16 | SI 16 | PV 16 | ES 21 | SI 21 | PV 21 |
| MZ  Twin 1 | ES 12 | .40  (.36, .43) | .23  (.20, .25) | .23  (.21, .26) | .22  (.19, .25) | .14  (.11, .17) | .14  (.10, .18) | .19  (.16, .23) | .13  (.09, .16) | .05  (.01, .09) |
|  | SI 12 |  | .38  (.35, .42) | .19  (.17, .22) | .12  (.09, .15) | .24  (.21, .27) | .15  (.11, .19) | .13  (.09, .16) | .17  (.14, .21) | .08  (.04, .12) |
|  | PV 12 |  |  | .56  (.53, .59) | .12  (.09, .15) | .15  (.12, .18) | .31  (.28, .34) | .13  (.10, .17) | .14  (.10, .17) | .16  (.12, .19) |
|  | ES 16 |  |  |  | .41  (.38, .45) | .21  (.18, .24) | .16  (.12, .20) | .28  (.24, .31) | .15  (.11, .18) | .09  (.05, .13) |
|  | SI 16 |  |  |  |  | .42  (.38, .45) | .18  (.14, .22) | .18  (.15, .22) | .30  (.27, .33) | .10  (.07, .14) |
|  | PV 16 |  |  |  |  |  | .46  (.41, .51) | .16  (.11, .20) | .18  (.14, .23) | .23  (.18, .27) |
|  | ES 21 |  |  |  |  |  |  | .34  (.30, .38) | .20  (.17, .23) | .12  (.08, .15) |
|  | SI 21 |  |  |  |  |  |  |  | .38  (.34, .42) | .06  (.03, .10) |
|  | PV 21 |  |  |  |  |  |  |  |  | .30  (.25, .34) |
|  |  | DZ Twin 2 | | | | | | | | |
|  |  | ES 12 | SI 12 | PV 12 | ES 16 | SI 16 | PV 16 | ES 21 | SI 21 | PV 21 |
| DZ  Twin 1 | ES 12 | .20  (.17, .23) | .13  (.11, .16) | .16  (.14, .19) | .12  (.09, .14) | .10  (.07, .12) | .09  (.05, .12) | .10  (.07, .13) | .10  (.07, .13) | .05  (.02, .08) |
|  | SI 12 |  | .21  (.18, .24) | .13  (.10, .15) | .07  (.05, .10) | .16  (.13, .18) | .09  (.05, .13) | .07  (.04, .10) | .12  (.09, .15) | .06  (.03, .09) |
|  | PV 12 |  |  | .34  (.31, .37) | .10  (.07, .13) | .09  (.07, .12) | .16  (.13, .20) | .11  (.08, .14) | .13  (.10, .15) | .10  (.07, .13) |
|  | ES 16 |  |  |  | .17  (.13, .20) | .11  (.09, .14) | .09  (.06, .13) | .15  (.12, .18) | .09  (.06, .12) | .05  (.02, .08) |
|  | SI 16 |  |  |  |  | .19  (.16, .23) | .12  (.08, .16) | .09  (.06, .12) | .12  (.10, .15) | .04  (.01, .07) |
|  | PV 16 |  |  |  |  |  | .21  (.16, .25) | .07  (.03, .11) | .07  (.03, .11) | .13  (.08, .17) |
|  | ES 21 |  |  |  |  |  |  | .19  (.15, .23) | .11  (.08, .14) | .06  (.03, .09) |
|  | SI 21 |  |  |  |  |  |  |  | .19  (.15, .23) | .05  (.02, .08) |
|  | PV 21 |  |  |  |  |  |  |  |  | .14  (.09, .18) |
| *Note*. We estimated correlations using the constrained saturated model in which means and within-person correlations were constrained to equality across zygosity, but within-pair correlations were symmetrical for MZ and DZ twins. Abbreviations: ES = emotional symptoms; SI = social isolation; PV = peer victimisation; 12 = 12 years old; 16 = 16 years old; 21 = 21 years old. | | | | | | | | | | |

Figure S1. Phenotypic Cross-Lagged Model Using the Full Peer Problems Scale (Sensitivity Analysis)


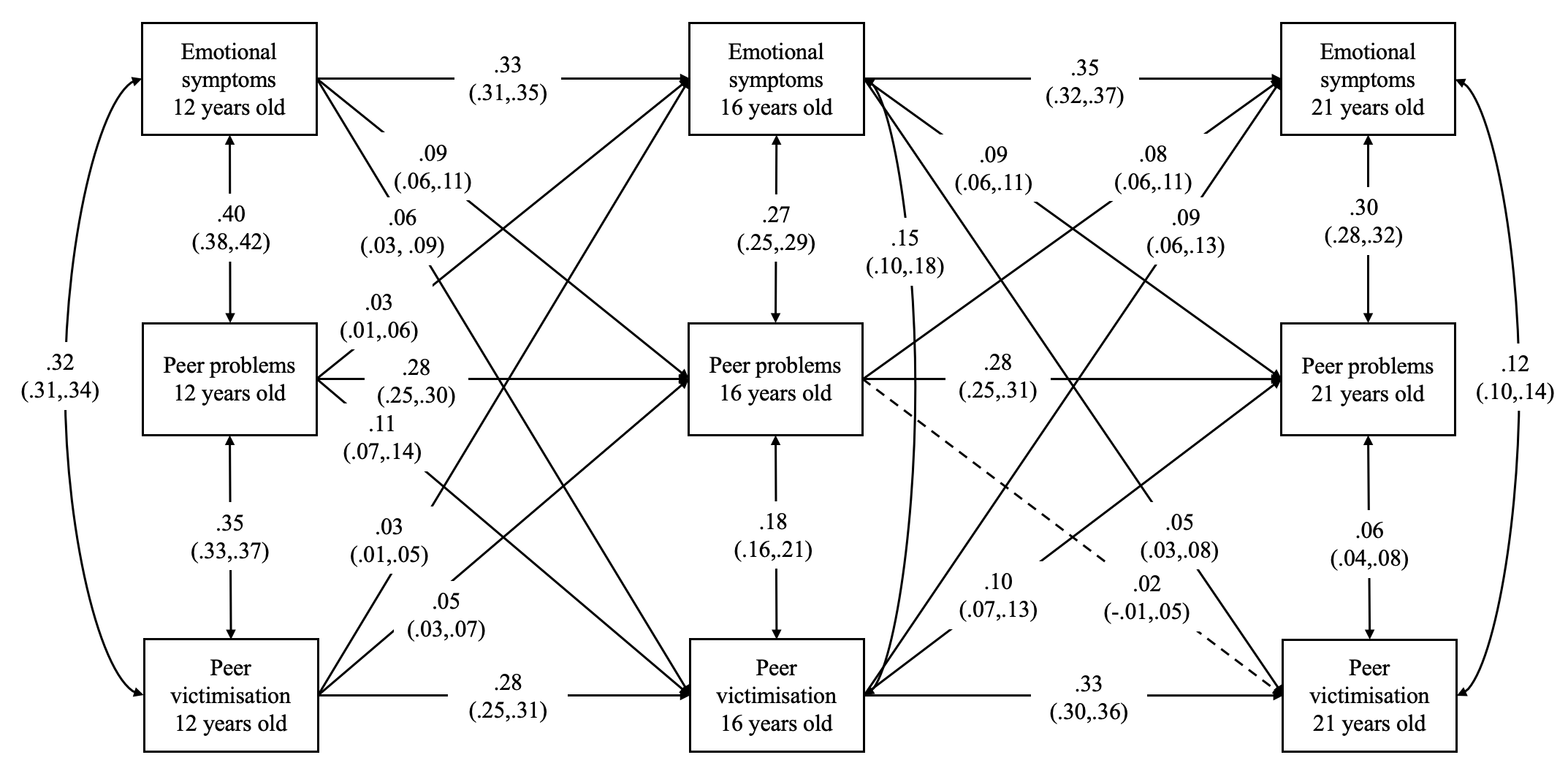


Figure S2. Genetic Cross-Lagged Model Using the Full Peer Problems Scale (Sensitivity Analysis)


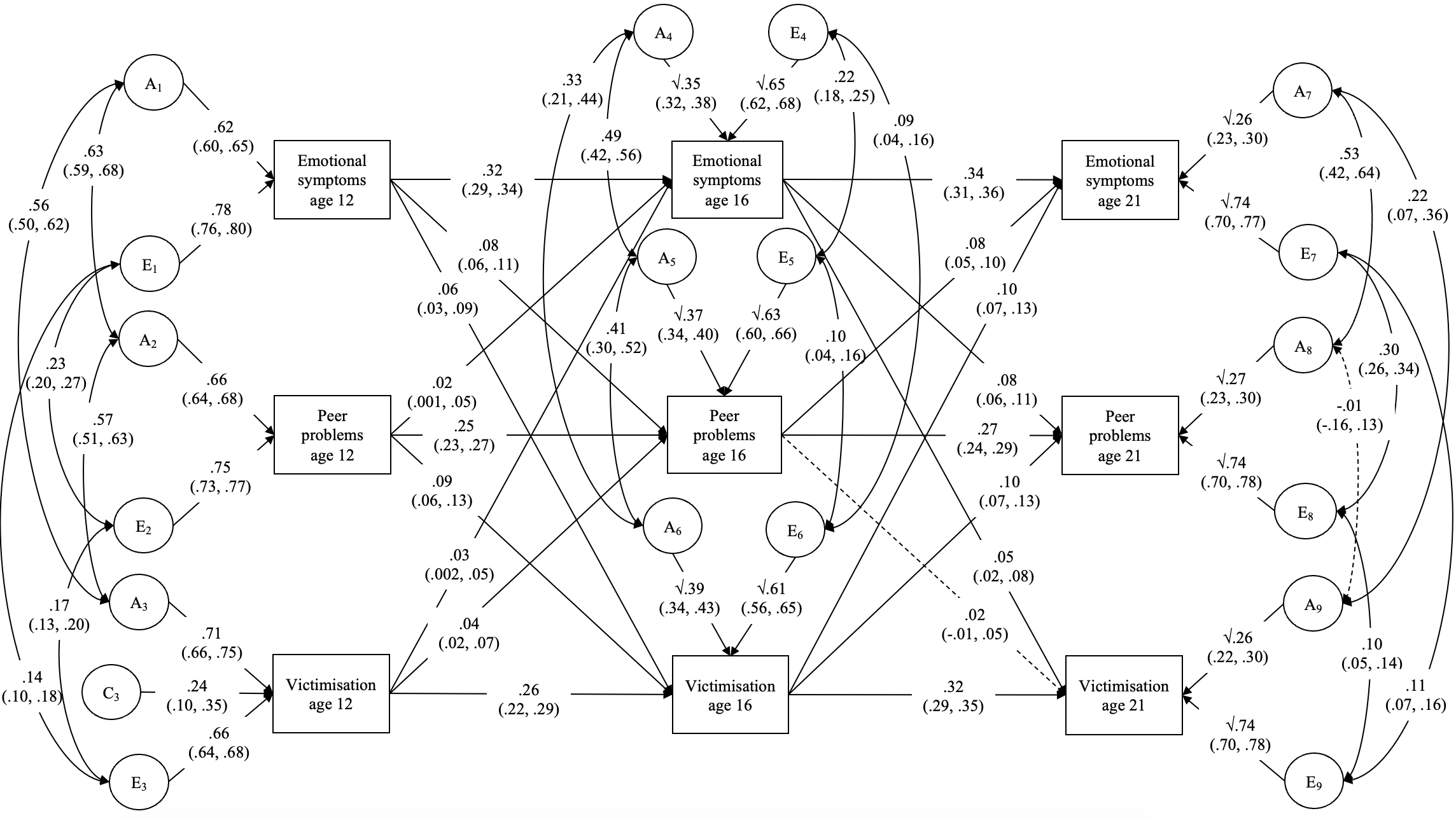


Figures S3. Multi-Group Phenotypic Cross-Lagged Model (Sex Differences)

A) Model for Females


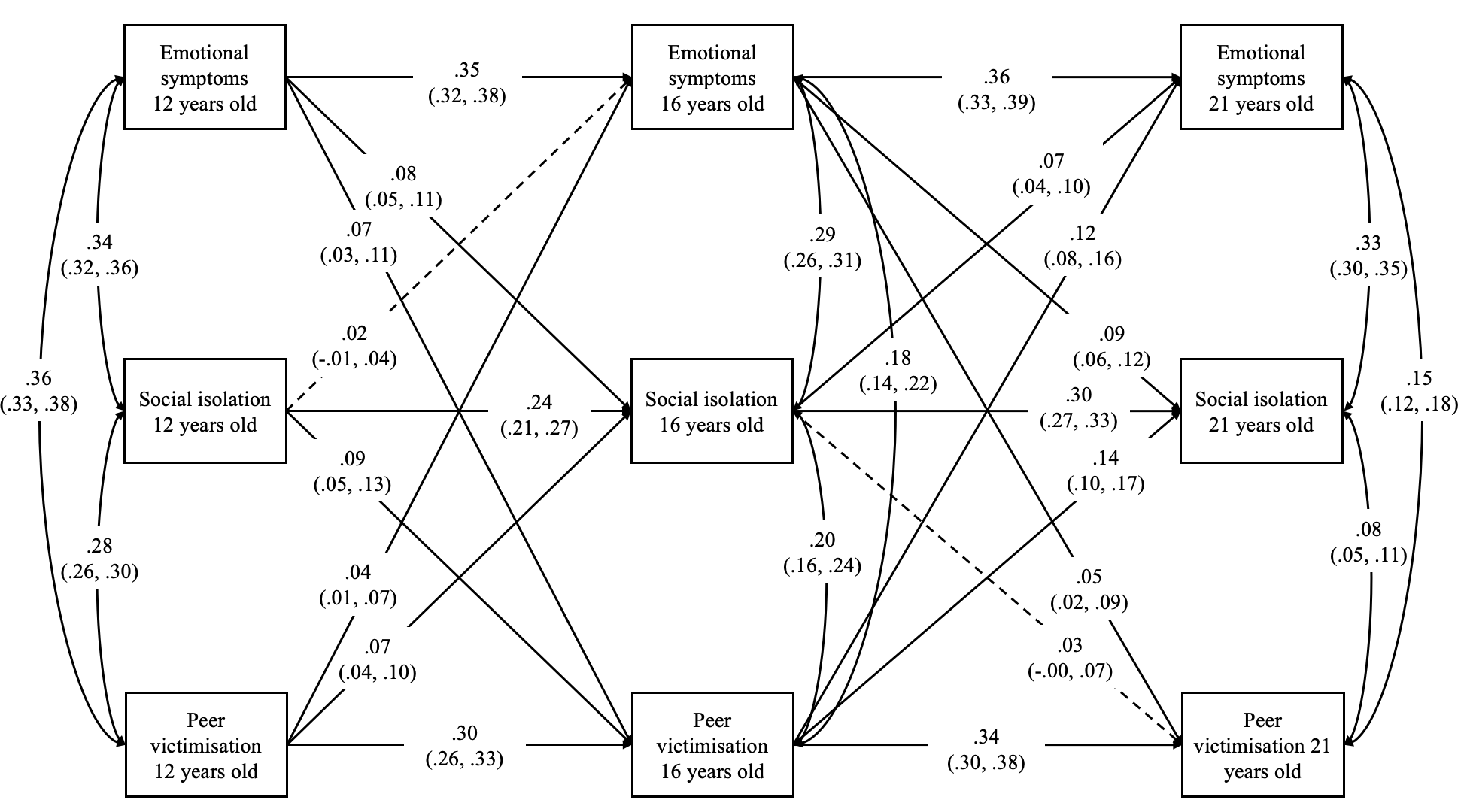


B) Model for Males


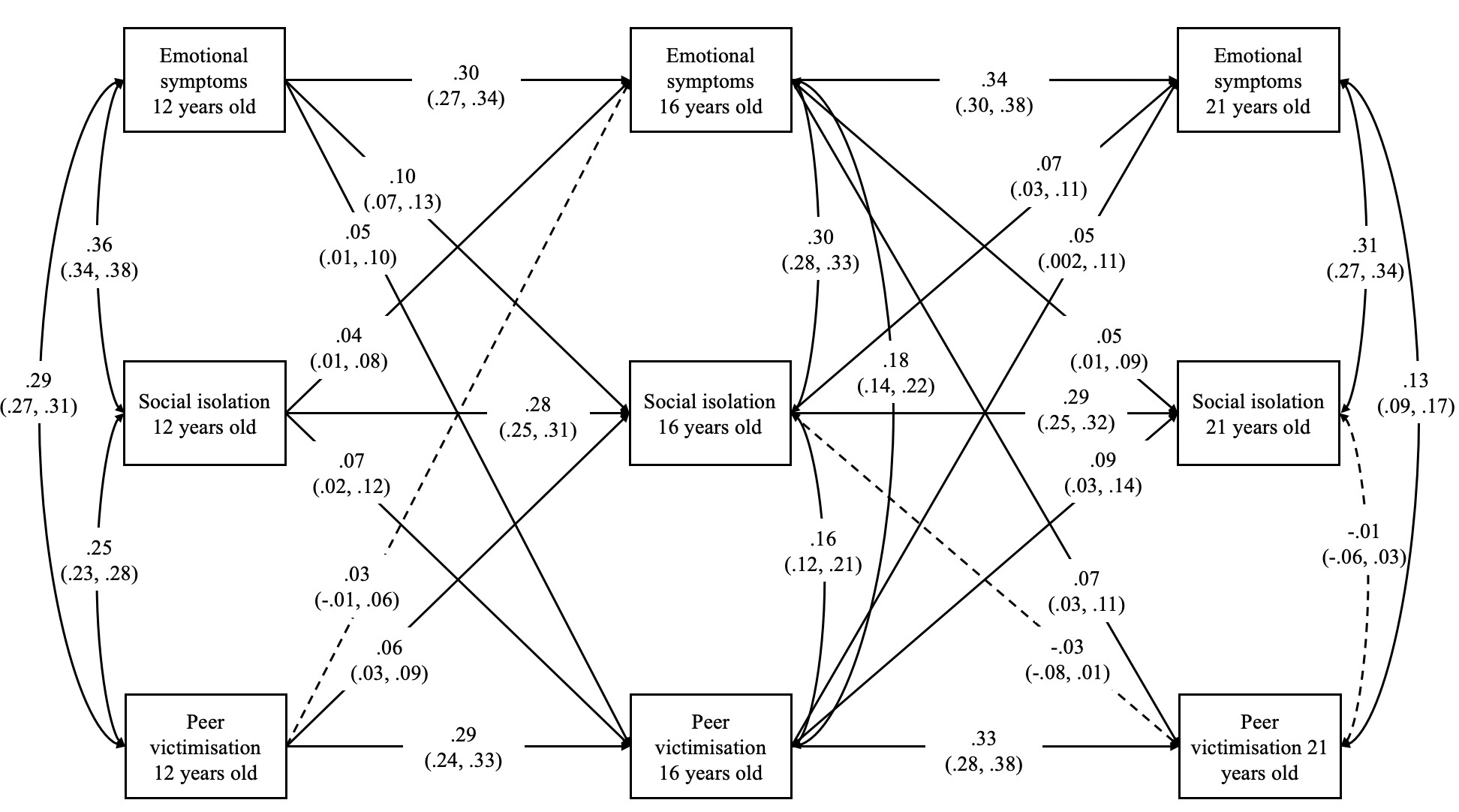


*Note*. This model showed significant improvement in fit, compared to a model in which all parameters were constrained to equality across sexes: Δdf = 37, ΔLL = 84.58, *p* < .001.
